# Neuromyelitis optica spectrum disorder with herpes simplex viral infection presenting with syndrome of inappropriate antidiuretic hormone: A case report

**DOI:** 10.1097/MD.0000000000035566

**Published:** 2023-10-27

**Authors:** Ji Yeon Chung, Chang Ju Lee, Jeong Bin Bong, Byoung-Soo Shin, Han Uk Ryu, Hyun Goo Kang

**Affiliations:** a Department of Neurology, Chosun University Medical School, Gwangju, South Korea; b Department of Neurology and Research Institute of Clinical Medicine of Jeonbuk National University, Jeonju, South Korea; c Biomedical Research Institute, Jeonbuk National University Medical School and Hospital, Jeonju, South Korea.

**Keywords:** area postrema, demyelination, hyponatremia, neuromyelitis optica spectrum disorder, syndrome of inappropriate antidiuretic hormone

## Abstract

**Rationale:**

Neuromyelitis optica spectrum disorder (NMOSD) is a demyelinating disease that causes lesions in areas with abundant aquaporin-4 (AQP4) channels, including the hypothalamus. Hypothalamic lesions can disrupt antidiuretic hormone regulation, resulting in hyponatremia due to syndrome of inappropriate antidiuretic hormone (SIADH). Various factors can trigger NMOSD, including viral infections. We report the case of a young female patient who presented with hyponatremia due to SIADH and was found to have bilateral hypothalamic lesions along with positive serum herpes simplex virus immunoglobulin M.

**Patient concerns:**

An 18-year old female patient presented with fever and nausea that had persisted for 5 days. Three days after hospitalization, the patient complained of blurred vision, hiccups, and excessive daytime sleepiness.

**Diagnosis:**

The patient hyponatremia was attributed to SIADH. Magnetic resonance imaging revealed bilateral lesions in the hypothalamus, and serum laboratory tests were positive for herpes simplex virus immunoglobulin M. On the 15th day of admission, the anti-AQP4 antibody test result was positive, leading to the diagnosis of NMOSD.

**Interventions:**

On the initial suspicion of herpes encephalitis, treatment with acyclovir was initiated. However, upon the confirmation of after anti-AQP4 antibody, the patient was additionally treated with a high-dose intravenous steroid for 5 days.

**Outcomes:**

The patient fever, nausea, visual disturbances, and other complaints improved within 1 week of initiating steroid treatment.

**Lessons:**

In young patients presenting with hyponatremia and suspected SIADH accompanied by neurological abnormalities, it is crucial to differentiate central nervous system diseases, including NMOSD, which can involve lesions in AQP4-abundant areas, such as the hypothalamus.

## 1. Introduction

Neuromyelitis optica spectrum disorder (NMOSD) is an inflammatory demyelinating autoimmune disease that causes lesions not only in the optic nerves and spinal cord, but also in the periventricular area where aquaporin 4 (AQP-4) channels are abundant.^[1]^ These AQP-4 channels are primarily distributed in the optic nerves, spinal cord, periventricular area, hypothalamus, subpial space, and area postrema.^[2]^ Depending on the lesion site, various initial symptoms can occur.^[3]^ Lesions in the hypothalamic region, specifically affecting the area responsible for antidiuretic hormone regulation, can lead to hyponatremia due to the syndrome of inappropriate antidiuretic hormone (SIADH).^[3]^ In addition, the hypothalamus plays in role in thermoregulation, and hyperthermia can occur if a lesion develops in hypothalamic area.^[4]^

There are various triggering factors for NMOSD, although not all have been fully elucidated. Some studies suggest the molecular mimicry associated with viral infections may affect aquaporin function, thereby leading to NMOSD.^[5]^ However, another study that measured viral antibodies in several NMOSD patients reported no significant correlation between viral infections and NMOSD.^[6]^ At present, it remains unclear whether the occurrence of viral infections and NMOSD is subsequent or consequent.

Herein, we report a young female patient who presented with nausea and fever and showed signs of SIADH. Lesions in the periventricular area, bilateral hypothalamus, area postrema, and spinal cord were confirmed using magnetic resonance imaging (MRI); concurrently, the test for serum herpes simplex virus (HSV) immunoglobulin M (IgM) antibodies was positive. A few days later, the patient was confirmed positive for AQP-4 antibodies, leading to a final diagnosis of NMOSD.

## 2. Case presentation

An 18-year-old female patient, who was usually healthy, presented to the emergency room with persistent fever and nausea for the last 5 days. The patient had no history of vaccination or infectious diseases within the previous 4 weeks. At the time of the visit, the patient had fever, with a body temperature was 37.8°C; blood pressure, heart rate, and respiration rate were within the normal range. The patient was alert and oriented and did not show any symptoms of cranial nerve palsies besides nausea. No signs of limb weakness, sensory abnormalities, or cerebellar dysfunction were observed. A routine blood test conducted in the emergency room revealed hyponatremia (serum sodium level, 114 mEq/L). Although the patient complained of a feeling of lack of energy and reduced food intake because of the nausea, there were no signs of dehydration (skin turgor and tongue dehydration). Serum osmolality had decreased to 242 mOsm/kg, urine osmolality was 317 mOsm/kg, and urine sodium level was 34 mg/dL. As a result, the patient was admitted to the endocrinology department for further evaluation and treatment under the suspicion of SIADH. The suspicion of SIADH was due to the patient signs of hypo-osmolality and hyponatremia, although the urine osmolality was not low. Thus, the possibility of cerebral salt wasting syndrome (CSWS) was excluded. Hypothyroidism and hypoadrenalism, which can cause hyponatremia, were normal. In addition, there were no specific findings related to rheumatoid factors.

**Figure 1.**
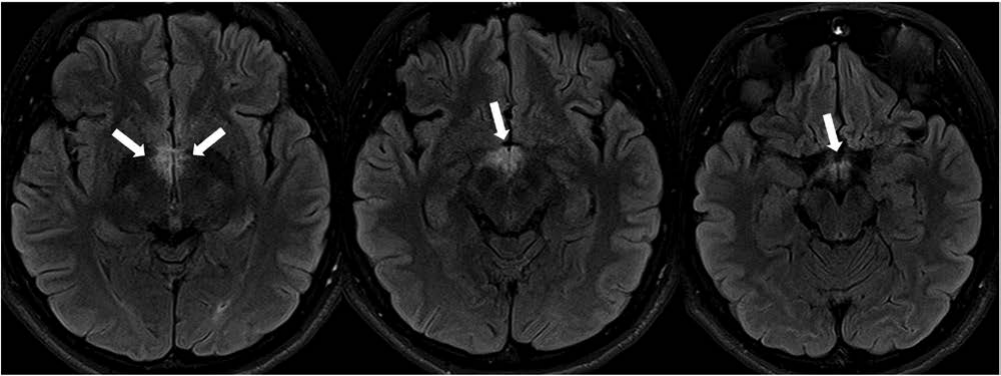
Brain magnetic resonance imaging (MRI) on the 3^rd^ d of admission. Fluid-attenuated inversion recovery imaging (FLAIR) showed symmetrical hyperintensities in the anteroinferomedial thalamus, hypothalamus, mamillary body.

The serum sodium level gradually improved, and by the second day of hospitalization, the sodium level recovered to a range close to normal (Na = 132 mEq/L). However, the mild fever and nausea persisted. On the third day of hospitalization, the patient complained of blurry vision, persistent hiccups, and daytime drowsiness. Follow-up neurological examinations showed no neurological abnormalities other than gaze-evoked nystagmus. Brain MRI was performed on the third day of hospitalization to differentiate the central lesions, and fluid-attenuated inversion recovery imaging showed symmetrical hyperintensities in the hypothalamus, mammillary body, periventricular region, hippocampus, area postrema, and around the fourth ventricle. No optic nerve lesions associated with visual disturbance were observed (Fig. 1). To differentiate between infectious causes that can affect the bilateral hypothalamus and limbic system, several infectious marker tests were conducted. The serum HSV IgM test showed a positive result with a value of 1.26 (normal range: 0–0.89). Herpes viral infection can invade the brain, causing bilateral lesions in the hypothalamus and limbic system. A cerebrospinal fluid (CSF) test was performed on the fourth day of hospitalization to differentiate between central nervous system infection, autoimmune disease, and demyelinating disease. The CSF appeared clear, with normal intracranial pressure, and showed elevated white blood cell count of 23/mm^3^, consisting entirely of lymphocytes. Both protein (29 mg/dL) and glucose (59 mg/dL) levels were normal, and the oligoclonal band test was negative. While waiting for the CSF HSV polymerase chain reaction (PCR) results, treatment with acyclovir was initiated due to the suspicion of herpes encephalitis.

**Figure 2.**
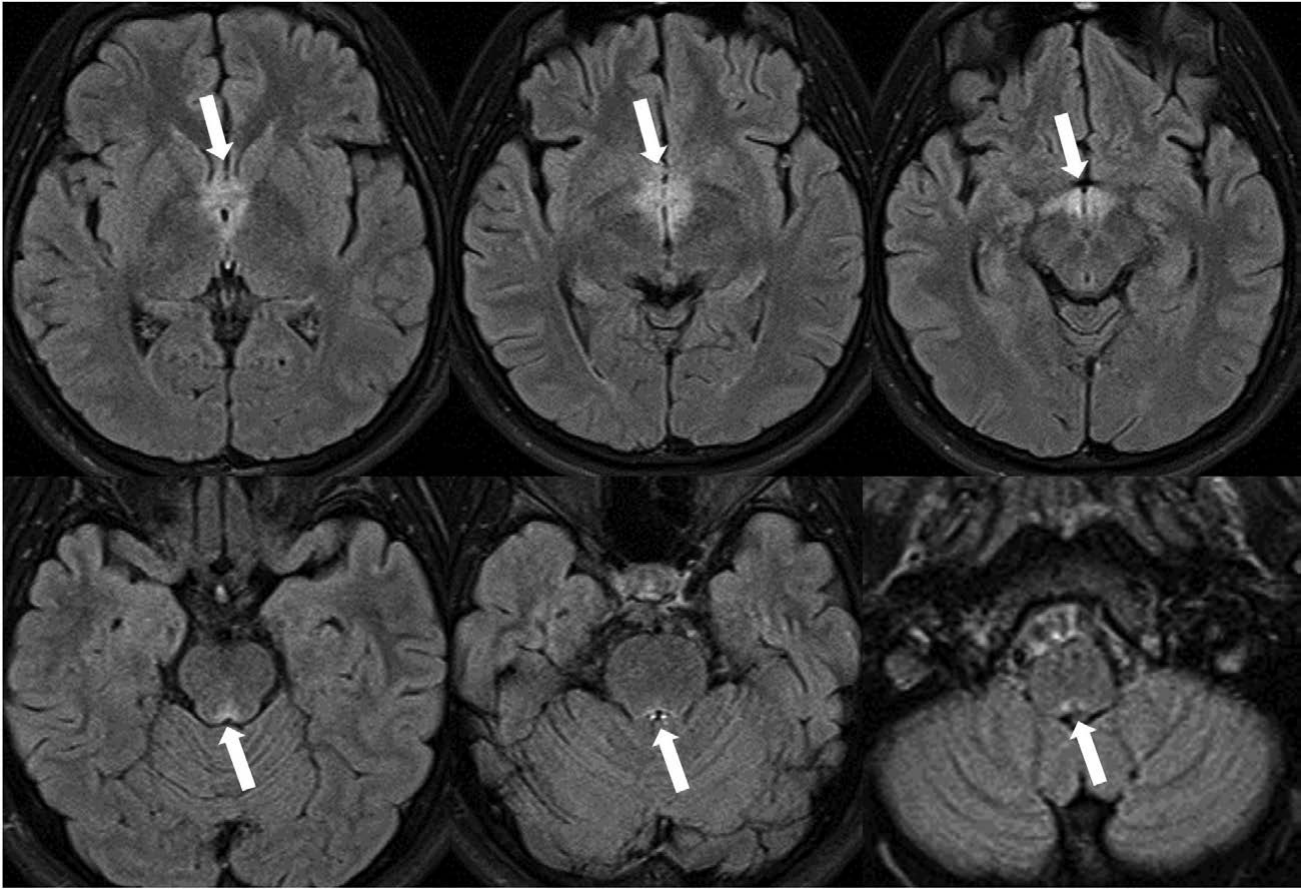
Brain magnetic resonance imaging (MRI) follow-up in the 2^nd^ wk of hospitalization. Fluid-attenuated inversion recovery (FLAIR) images show the hyperintense lesions in the hypothalamus, periaqueductal area, periependymal surface of fourth ventricle and area postrema. The hyperintensity lesions are more pronounced compared with the initial brain MRI.

**Figure 3.**
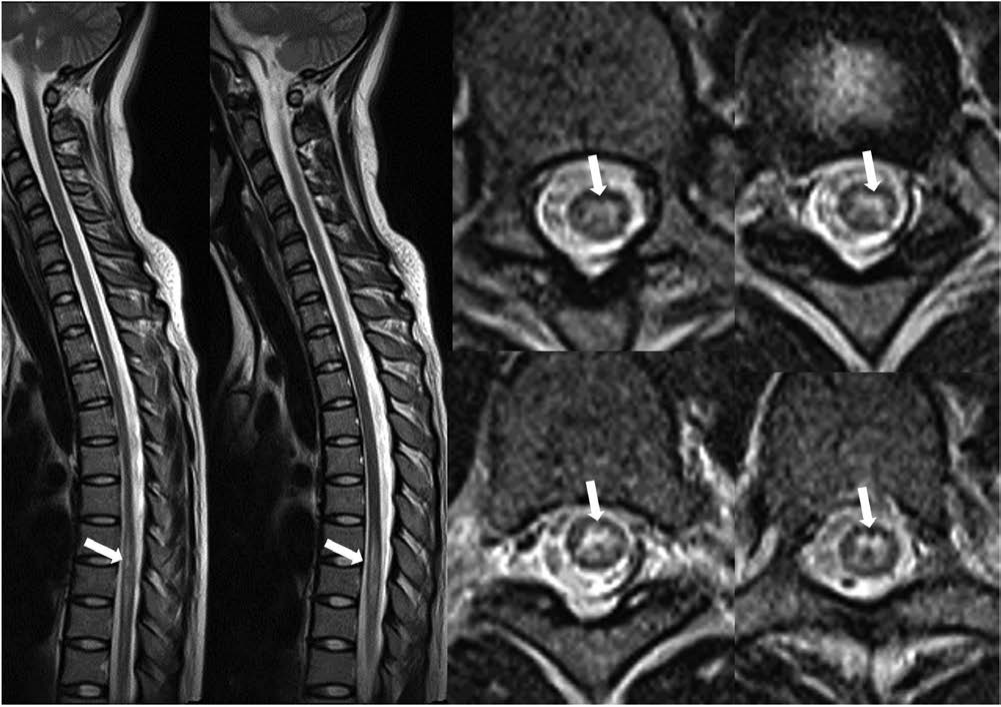
Spinal cord magnetic resonance imaging (MRI) in the 2^nd^ wk of hospitalization. Longitudinal extended transverse myelitis (LETM) lesion of T2 hyperintensity in thoracic spinal cord T7–8-9 level.

Hiccups improved the day after treatment initiation; however, symptoms of nausea, vomiting, excessive drowsiness, and visual blurring continued without improvement. A slight fever of 37.7°C persisted for 1 week after hospitalization. There was no limb weakness or sensory abnormality and no Lhermitte sign was observed. Approximately 1 week after treatment initiation, nausea, vomiting, and excessive drowsiness markedly improved, and the serum HSV IgM titer turned negative on the seventh day of treatment. As the CSF HSV PCR result was negative, acyclovir treatment was discontinued. Although the symptoms of blurred vision persisted, the patient did not complain of ocular pain, visual field defects, or abnormalities in color perception. An immunofluorescence assay for APQ4 antibodies was conducted to differentiate NMOSD, and AQP-4 antibody was confirmed to be positive at 1:000 (intensity 3+) 15 days after hospitalization. In the follow-up brain MRI performed on the second week of hospitalization, the contrast-enhanced lesions seen on the initial MRI became more pronounced (Fig. 2). Although there were no related symptoms, such as limb weakness or bowel and bladder dysfunction, additional whole-spine MRI T2 contrast-enhanced imaging showed edematous longitudinal extensive transverse myelitis lesions with high signal intensity at the T7–8-9 level (Fig. 3). The patient was diagnosed with NMOSD based on persistent neurological abnormalities, brain and spinal cord imaging findings specific for NMOSD, and antibody test results. The patient blurred vision improved within 1 week of additional administration of high-dose intravenous steroids. Subsequently, the treatment was switched to oral steroids and gradually tapered off. As the time of this report, the patient is being treated with mycophenolate mofetil and observed regularly in outpatient follow-up visits.

## 3. Discussion

In the present case, an 18-year-old female patient presented to the emergency room with initial symptoms of nausea and fever, and laboratory test results showed hyponatremia. SIADH was identified as the cause of hyponatremia; therefore, further evaluation was conducted to determine the cause of SIADH and correct sodium levels. However, the patient complained of additional symptoms, including hiccups, sleepiness, and blurring of vision. Brain MRI revealed lesions in the periventricular region, hypothalamus, and area postrema. In addition, spinal cord MRI confirmed the presence of longitudinal extensive transverse myelitis lesions at the thoracic (T7–8-9) level. The test for serum HSV IgM antibodies was positive, and CSF examination revealed an increase in the number of leukocytes. Considering the possibility of HSV encephalitis, which is known to be associated with SIADH, we waited for the results of the CSF HSV PCR while initially administering antiviral agents. However, the CSF HSV PCR results were confirmed to be negative, and the AQP-4 antibody was positive. The final diagnosis was NMOSD and the treatment plan was modified accordingly.

The characteristic brain lesions of NMOSD typically occur in areas abundant in AQP-4, including the periependymal regions surrounding the lateral ventricles, and the cerebral aqueduct.^[7]^ These lesions are the anterior border of the thalamus, hypothalamus, and midbrain.^[7]^ NMOSD frequently invades the area postrema and nucleus tractus solitarius, the dorsal medulla oblongata adjacent to the fourth ventricle.^[7]^ When periependymal lesions surrounding the lateral ventricles are affected in NMOSD, SIADH, symptoms such as narcolepsy, and changes in body temperature can occur. If the dorsal medulla is affected, hiccups, nausea, vomiting, or nystagmus may occur. Among the areas with high AQP-4 expression, the area postrema, a chemo-sensitive vomiting center, can be the first attack point of NMOSD.^[8]^ attacks on AQP-4 in the paraventricular nucleus regions of the hypothalamus are susceptible to complement-induced damage.^[3,9]^ Some lesions in this area are associated with endocrinopathy, which can lead to SIADH, a condition characterized by dysregulation of antidiuretic hormones.^[3,9]^ SIADH is the most common cause of hyponatremia.^[10]^ Meanwhile, SIADH is needs to be differentiated from CSWS. Although it is challenging to clinically distinguish SIADH from CSWS, if the volume of extracellular fluid is within normal limits, the condition is more likely to be SIADH than CSWS. Differential diagnosis is required for herpes encephalitis in cases involving the bilateral symmetric thalamus and limbic areas. In our patient, the initial symptoms were fever and nausea, with the gradual development of dizziness, hiccups, and blurred vision. Furthermore, blood tests revealed hyponatremia and positive serum HSV IgM antibody findings, and brain imaging revealed symmetrical hyperintensities in the bilateral thalamus, brainstem, and area postrema. Therefore, based on the initial laboratory results, antiviral treatment was initiated, considering HSV encephalitis involving the hypothalamus accompanied by SIADH.

The patient in this case report initially presented with nausea, fever, and hyponatremia, suggesting SIADH in blood tests. Therefore, an initial treatment for SIADH was administered. Subsequently, gradual additional neurological abnormalities and positive laboratory findings were confirmed and additional sequential treatments were initiated for suspected diseases. Previous studies have proposed several hypotheses regarding the association between hyponatremia, SIADH, HSV, and NMOSD.^[5,6]^ These conditions may have causal relationships, but there is also a possibility that they occur independently and concurrently. Turco et al^[5]^ hypothesized that a viral infection might trigger NMOSD through molecular mimicry affecting aquaporin function. Moreover, Turco et al^[6]^ tested for potentially associated viral antibodies in patients with NMOSD but found no significant association. It remains unclear whether this was a subsequent or a consequent effect.

This case suggests that in young patients with hyponatremia suspected to have SIADH accompanied by neurological abnormalities, it is necessary to differentiate central nervous system diseases. It is essential to check for hypothalamic lesions associated with antidiuretic hormone regulation. In particular, since AQP-4 is highly expressed in the hypothalamic region, considering the possibility NMOSD due to anti-AQP4 antibodies can be helpful in the initial diagnosis and treatment. In addition, if brain lesions involve the thalamic region and are bilaterally symmetrical, NMOSD may be triggered by viral infections. Therefore, when a concomitant viral infection is suspected, serum and CSF tests should be conducted. If specific viral antibodies are confirmed, a combination of antiviral therapies should be considered.

## Author contributions

**Conceptualization:** Ji Yeon Chung, Hyun Goo Kang.

**Data curation:** Ji Yeon Chung, Chang Ju Lee.

**Formal analysis:** Chang Ju Lee.

**Investigation:** Ji Yeon Chung.

**Methodology:** Byoung-Soo Shin.

**Project administration:** Han Uk Ryu, Hyun Goo Kang.

**Software:** Chang Ju Lee.

**Supervision:** Jeong Bin Bong, Byoung-Soo Shin, Han Uk Ryu, Hyun Goo Kang.

**Validation:** Byoung-Soo Shin, Han Uk Ryu.

**Writing – original draft:** Ji Yeon Chung.

**Writing – review & editing:** Ji Yeon Chung, Jeong Bin Bong, Hyun Goo Kang.
